# Niacin and Stroke: The Role of Supplementation and Emerging Concepts in Clinical Practice, a Narrative Review

**DOI:** 10.3390/cimb47060400

**Published:** 2025-05-28

**Authors:** Alan D. Kaye, Grant D. Coffman, Sydney A. Mashaw, Austin S. Thomassen, Kalob M. Broocks, Ahmed I. Anwar, Shahab Ahmadzadeh, Sahar Shekoohi

**Affiliations:** 1 Departments of Anesthesiology and Pharmacology, Toxicology, and Neurosciences, Louisiana State University Health Sciences Center at Shreveport, Shreveport, LA 71103, USA; 2 School of Medicine, Louisiana State University Health Sciences Center at Shreveport, Shreveport, LA 71103, USAsam008@lsuhs.edu (S.A.M.);; 3 Department of Psychology, Quinnipiac University, Hamden, CT 06518, USA; 4 Department of Anesthesiology, Louisiana State University Health Sciences Center at Shreveport, Shreveport, LA 71103, USA

**Keywords:** niacin, stroke, cardiovascular disease, stroke prevention, low-density lipoproteins, triglycerides, high-density lipoproteins

## Abstract

Niacin therapy has been a mainstay in traditional secondary prevention of stroke. Supplemental use of niacin has been used to improve lipid panel markers, including lowering low-density lipoproteins and triglycerides, and raising high-density lipoproteins, which have been associated with lower risk for stroke. This supplementation has been supported by earlier studies regarding niacin and its role in reducing cardiovascular risk. However, recent studies have called into question the efficacy of niacin therapy and some studies even show negative effects that are associated with taking supplemental niacin. In the present investigation, review of niacin-mediated benefits to cardiovascular disease and newer research suggesting that niacin may be ineffective with the potential of adverse side effects are summarized. This review further highlights the need for more population-level studies regarding the effects of supplemental niacin use to provide a scientific basis for its therapeutic role in cardiovascular disease, including improvement in lipid panel markers and stroke prevention.

## 1. Introduction

Niacin, also known as vitamin B3, is a water-soluble nutrient essential for various physiological processes. It encompasses a group of compounds, including nicotinic acid (pyridine-3-carboxylic acid), nicotinamide (niacinamide or pyridine-3-carboxamide), and derivatives such as nicotinamide riboside [1]. The Food and Nutrition Board (FNB) of the National Academies of Sciences, Engineering, and Medicine suggests a daily intake of 16 mg of niacin for men and 14 mg for non-pregnant women as the recommended dietary allowance (RDA) [2]. Niacin can be obtained naturally from dietary sources such as red meat, fish, poultry, eggs, whole grains, legumes, and whole-grain cereals [3]. Supplemental niacin is available as nicotinic acid or nicotinamide in over-the-counter or prescription forms [4]. Unlike dietary niacin, which is naturally bound to proteins and gradually absorbed during digestion, supplements provide a more concentrated dose, often used to manage specific health conditions such as dyslipidemia or niacin deficiency [5]. However, high doses from supplementation can exceed nutritional needs, potentially causing side effects such as skin flushing, dizziness, and hypertension [1].

Niacin serves as a precursor to the pyridine nucleotides NAD (nicotinamide adenine dinucleotide) and NADP (nicotinamide adenine dinucleotide phosphate), which are essential cofactors in cellular metabolic redox reactions. These molecules play a critical role in processes where substrates undergo oxidation or reduction, supporting energy production and overall metabolic function [6].

Beyond involvement in many fundamental biochemical processes, niacin plays a crucial role in maintaining endothelial function, a key factor in maintaining optimal vascular health. It promotes vasodilation by increasing the production of prostacyclin (PGI2), resulting in improved blood flow [7]. It also enhances nitric oxide (NO) bioavailability, facilitating vasodilation via smooth muscle relaxation in blood vessel walls [8]. While this nitric oxide enhancement has not been established to significantly reduce cerebrovascular events, theoretically its role in regulating cerebral blood flow may help maintain healthy brain vasculature [9]. Niacin also improves lipid profiles, raising high-density lipoprotein (HDL) cholesterol and lowering low-density lipoprotein (LDL) cholesterol and triglycerides (TGs), thereby reducing the burden on the endothelium and potentially preventing or slowing progression of atherosclerosis [10,11].

Niacin, or vitamin B3, has been historically utilized to manage dyslipidemia and decrease the risk of cardiovascular events, including strokes. Stroke is a leading cause of death and disability worldwide, with ischemic stroke accounting for around 87% of all cases [12]. The primary cause of ischemic stroke is the blockage of blood flow to the brain due to atherosclerotic plaque formation or thromboembolism. Key risk factors include hypertension, diabetes, smoking, dyslipidemia, and a sedentary lifestyle. Dyslipidemia, defined by abnormal levels of lipids in the blood, is a major modifiable risk factor for stroke and other cardiovascular events. Enhanced levels of low-density lipoprotein (LDL) cholesterol and low levels of high-density lipoprotein (HDL) cholesterol are associated with atherosclerosis and stroke risk. Niacin blocks the release of free fatty acids from adipose tissue, resulting in a reduction in triglyceride levels and a decrease in the conversion of HDL cholesterol to LDL cholesterol [13]. These lipid-modulating properties of niacin have played a supportive role in stroke prevention strategies.

In the healthy state, the endothelium, which lines the blood vessels, has an essential role in maintaining vascular homeostasis by regulating blood flow, promoting vasodilation, and preventing excessive clotting [14]. The reduced availability of NO results in vasoconstriction, exacerbating the blockage created by the plaque and further limiting blood flow to the brain [15].

In addition to these changes, inflammation plays an important role in the progression of atherosclerosis and the pathophysiology of stroke [16]. As the plaque forms and grows, it triggers an inflammatory response within the arterial wall. Immune cells, such as macrophages, infiltrate the site and release pro-inflammatory cytokines and other mediators. The chronic inflammation within the vessel wall further damages the endothelium and promotes the instability of the plaque. When the plaque ruptures, its thrombogenic core is exposed to the bloodstream, leading to clot formation. The resulting thrombi lead to complete obstruction of blood flow, causing ischemic stroke [17,18]. Preventative strategies targeting lipid control, vascular health, and inflammation are critical in reducing stroke risk [19,20,21].

The role of niacin in cardiovascular disease (CVD) prevention has been well documented over the past several decades [22]. Clinical trials in the late 20th century, such as the Coronary Drug Project, showed that niacin had favorable effects on lipid profiles and endothelial function [23,24]. It was frequently used in conjunction with statins to achieve more pronounced lipid-lowering effects [25]. As a result, niacin became a mainstay in the management of dyslipidemia and the prevention of CVD [26]. While niacin has been used as a prophylactic for cerebrovascular disease, there is little to no evidence that it may be useful for recovery from a recent stroke [27].

Due to new evidence in recent years, the clinical use of niacin in cardiovascular disease (CVD) prevention has been reevaluated due to emerging evidence questioning its long-term efficacy [28]. Large-scale trials, including the AIM-HIGH and HPS2-THRIVE studies, found no significant decrease in major cardiovascular events with niacin therapy, despite improvements in lipid profiles [29,30]. Furthermore, recent studies suggest that the terminal breakdown products of excess niacin, 2PY and 4PY, may contribute to increased CVD risk by promoting vascular inflammation. This inflammatory response could accelerate atherosclerosis, raising concerns about niacin’s overall impact despite its positive effect on lipid profiles [31]. These results may lead to a reassessment of niacin’s role in modern treatment regimens, both as a standalone therapy and when combined with statins. The present investigation, therefore, summarizes the traditional use of niacin for stroke prevention, emerging evidence against its efficacy, and clinical considerations that impact its use in stroke risk management.

## 2. The Traditional Use of Niacin for Stroke Prevention

### 2.1. Niacin and Lipid Modulation

Niacin exerts its effects on lipid metabolism through several mechanisms. It inhibits lipolysis in adipose tissue, reducing the free fatty acid supply to the liver and, as a result, the hepatic production of very low-density lipoprotein (VLDL) and LDL. In addition to this, niacin enhances HDL levels by decreasing its hepatic catabolism [32]. These effects collectively contribute to its ability to modulate lipid profiles in a beneficial way. Although niacin’s effect on LDL is less potent compared to statins, it can reduce LDL cholesterol levels by 10–15% [33]. This effect is slightly higher than the effect of fibrates on LDL reduction. Lower LDL levels are directly associated with reduced atherosclerotic plaque formation and stabilization of existing plaques, reducing the risk of thrombotic events. Niacin also lowers triglyceride levels by 20–50%, further mitigating atherosclerosis risk. Enhanced triglycerides are an independent risk factor for both stroke and cardiovascular disease [34].

Niacin is one of the most effective agents for increasing HDL cholesterol levels, often raising them by 15–35% [35]. Elevated HDL levels are inversely correlated with cardiovascular and cerebrovascular events [36]. By improving the lipid profile, niacin theoretically reduces the progression of atherosclerosis, decreases plaque instability, and minimizes the likelihood of stroke.

### 2.2. Niacin Supplementation vs. Dietary Niacin for Stroke Prevention

Niacin can be obtained from dietary sources and supplementation. The therapeutic doses of niacin used for lipid modulation (1–2 g per day) far exceed the amounts typically obtained through diet [37]. Dietary niacin is essential for basic cellular metabolism and energy production, but does not significantly impact lipid profiles at dietary levels. Supplemental niacin, particularly in extended-release formulations, is required to achieve the lipid-modulating effects necessary for stroke prevention. However, high doses (greater than 2 g per day) of niacin supplementation are associated with side effects, such as flushing, gastrointestinal distress, and, in rare cases, hepatotoxicity. The use of niacin in clinical practice needs consideration of the risk-to-benefit ratio [38].

### 2.3. Niacin in Combination with Statins for Lowering Stroke Risk

Statins are the primary pharmacological agents for managing dyslipidemia and reducing cardiovascular risk. They inhibit HMG-CoA reductase resulting in a significant reduction in LDL cholesterol and a modest enhancement in HDL. Combining niacin with statins has been explored to amplify both of their lipid-modulating effects. Early studies suggested that the niacin-statin combination might provide additive benefits by addressing different aspects of dyslipidemia. For instance, while statins excel at reducing LDL cholesterol, niacin’s ability to increase HDL levels complements this effect [25]. The potential synergistic effect could theoretically lead to more substantial reductions in stroke and cardiovascular events via increased HDL levels. However, recent large-scale trials have questioned the clinical utility of this combination.

### 2.4. AIM-HIGH Trial

The AIM-HIGH trial (2011) evaluated the addition of extended-release niacin to statin therapy in patients with established cardiovascular disease and low HDL levels. Despite significant improvements in HDL levels, this study found no significant benefit of the addition of niacin in the reduction in cardiovascular events, including strokes, in patients with well-controlled LDL cholesterol levels, of less than 70 mg/dL. Their results show that patients on combination niacin-statin therapy saw a greater incidence of ischemic stroke, although nonsignificant, and describe this phenomenon as a “play of chance”. This finding, however, sparked an investigation into the role of niacin in ischemic stroke prevention [39].

### 2.5. HPS2-THRIVE Trial

Soon following the AIM-HIGH trial, the HPS2-THRIVE trial assessed the combination of extended-release niacin and laropiprant, a prostaglandin D2 receptor antagonist, with statins. This study found a higher incidence of new-onset diabetes, infection, and bleeding in patients treated with the niacin and laropiprant combination. However, these adverse effects cannot be fully attributed to niacin. This study did, however, raise further questions about the efficacy of niacin for lipid modification [30].

These trials, imperfect as they are, do underscore the challenges of translating niacin’s lipid-modulating effects into tangible cardiovascular benefits. While niacin’s abilities to raise HDL and lower LDL and triglycerides are well documented, its role in stroke prevention is less clear in the era of potent LDL-lowering therapies such as statins and PCSK9 inhibitors. As dyslipidemia is only one component of stroke risk, treatment for this condition still leaves patients open to other factors, such as endothelial dysfunction, coagulation abnormalities, and inflammation [40]. These factors are not well addressed by niacin supplementation.

Additionally, the side-effect profile of niacin, including flushing, dose-dependent hepatitis, and gastrointestinal upset, limits its tolerability and use [41]. The widespread use of more potent and effective drugs has diminished the relative contribution of niacin to cardiovascular and cerebrovascular risk reduction. At this point in pharmacological development, the limited benefits of niacin are not always worth the added side effects. Further research is needed to clarify niacin’s role in modern cardiovascular care.

## 3. The Case Against Niacin for Stroke Prevention

### 3.1. Considerations for Niacin Increasing Stroke Risk

Niacin has been regularly used for the treatment of high cholesterol and prevention of cardiovascular disease (CVD) for a multitude of years. Theoretically, niacin could play a role in stroke prevention since niacin blocks the release of free fatty acids from adipose tissue, resulting in a reduction in triglyceride levels and a decrease in the conversion of HDL cholesterol to LDL cholesterol. New research, as well as old, has brought to light its adverse effects and how it could possibly lead to an increased risk of stroke. While still unknown, there is a possibility that it may be due to niacin’s effects on blood pressure, glucose metabolism, its use in patients with pre-existing cardiovascular conditions, types of supplementations, and its terminal metabolites [42].

### 3.2. Niacin’s Metabolism Regulations

A major known side effect of niacin is its effect on glycemic metabolism. Niacin is a precursor of NAD^+^, a metabolite that exerts some of its effects on glycemic control and metabolism [43,44]. The major downstream mediator of glucose and lipid metabolism through NAD^+^ is the NAD^+^-dependent protein deacetylase (SIRT1). It is mainly through SIRT1 that NAD^+^ can enhance the risk of metabolic complications such as insulin resistance that can result in the development of metabolic syndrome and type 2 diabetes [43,45]. Niacin has been shown to increase fasting glucose in patients with or without diabetes mellitus; most of these increases are transient and will decrease with discontinuation of niacin. Increasing doses of extended-release niacin were also correlated to worsening glycemic control, while low doses seemed to be tolerated better and could be controlled with anti-diabetic therapies [46]. Also, niacin is associated with an increased risk of the development of diabetes when used by itself or in combination with laropiprant or statin therapies, due to niacin contributing to insulin resistance and statins impairing insulin sensitivity and secretion [42]. While many of niacin’s glycemic side effects are minimal or can be controlled with medication, it is still an important adverse effect that can lead to the development of additional problems over time.

### 3.3. Fluctuations in Blood Pressure

The effects niacin has on lowering blood pressure (BP) differ depending on acute or chronic treatment. The exact mechanism of how niacin exerts its effect on blood pressure is unclear, but several theories have been proposed. With acute administration of niacin, it is understood that blood pressure drops through acute vasodilatory effects [47,48]. How much blood pressure fluctuates with niacin administration also depends on if the patient is normo- or hypertensive. In normotensive patients, there was very little change in blood pressure that was maintained through an increase in heart rate and decreases in systemic vascular resistance (SVR), stroke volume (SV), and vascular compliance; where in hypertensive patients there were significant decreases in systolic BP (SBP), diastolic BP (DBP), pulse pressure (PP), (SVR), (SV), and mean arterial pressure (MAP) [48]. Chronic use of niacin is believed to cause changes in endothelial function and lipid impact to fluctuate blood pressure [47,48,49]. In cultured human aortic endothelial cells (HAEC), niacin was found to inhibit radical oxygen species (ROS) production and LDL oxidation, VCAM-1 and MCP-1 expression, monocyte adhesion, and NF-kB activation [50]. It was previously believed that niacin also increased cutaneous vasodilation through PGD2 type 1 (PD1) receptors; however, a study showed that might not be the case when laropiprant did not increase or decrease the effect of blood pressure during chronic niacin treatment [48]. While the majority of research shows niacin’s vasodilatory effect, a new study showed that an increase in dietary niacin was being associated with new-onset hypertension in Chinese adults [51]. This study showed an inflection point at 15.6 mg/d; patients taking under that amount had a decrease in new-onset hypertension where patients exceeding that amount had an increase [51]. More research needs to be conducted in this area to determine why.

### 3.4. Terminal Metabolites 2PY and 4PY

**Figure 1:**
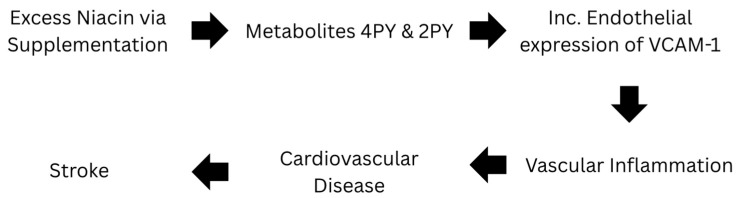
Pathway of Excess Niacin in Relation to Stroke.

Niacin itself is known to mainly cause vasodilation and have anti-inflammatory properties [47,48,49,50,52]. However, a recent study has found that its metabolites 2PY and 4PY are associated with increased inflammation and major adverse cardiovascular events (MACE) [31]. Specifically, individuals with 4PY levels in the top 25% had 1.6 to 2 times the risk of major cardiac events over the next three years compared to those with lower levels [31]. With 4PY being the major contributor, it is biologically active promoting inflammation and leukocyte adhesion. This metabolite directly increases endothelial cell activation and expression of VCAM-1, in both protein and RNA [31]. These products, 2PY, 4PY, and VCAM-1, are all associated with residual cardiovascular event risk and linked to risk of myocardial infarction (MI), stroke, and death [31]. These terminal metabolites could be responsible for the “niacin paradox”. Figure 1 shows the metabolic pathway for excess niacin and its relationship with stroke. 

### 3.5. The Role of Niacin in Patients with Pre-Existing Cardiovascular Disease

Niacin has been used in clinical practice for a long time, but studies are showing that patients taking it with pre-existing cardiovascular disease still retain or even have increased risk of serious cardiovascular conditions [28,42,49,53,54,55]. In relation to stroke, the HPS2-THRIVE trials showed that when compared to placebo, niacin-laropiprant had no significant effect on the incidence of presumed ischemic or hemorrhagic strokes [56]. This same study also found that adding extended-release (ER) niacin and laropiprant to a statin therapy did not have any reduction or benefit in cardiovascular events, but found other serious adverse effects like new-onset diabetes, bleeding, and infection [40]. Another major study, AIM-HIGH, found an increased number of ischemic strokes in patients with established cardiovascular disease taking simvastatin and extended-release niacin [39,56]. The study could not find a specific link between extended-release niacin and ischemic stroke; however, it should still warrant worry, and additional studies should be conducted to determine a possible link.

### 3.6. Dietary Niacin

Niacin is found in a lot of ordinary foods like meat, fish, legumes, nuts, and others. It can be consumed through our diet directly as nicotinic acid or synthesized from tryptophan [45]. Dietary niacin needs are almost always met through a typical diet and a need for extra supplementation is rarely needed. There are hardly ever side effects from ingesting too much niacin in daily diets; in fact, it typically has helpful regulation to lipid metabolism and anti-inflammatory properties [51]. However, a recent study in Chinese adults found that having too much dietary niacin was increasing the risk of developing new-onset hypertension [51]. More research needs to be conducted in this area to determine the impact of ingesting increased amounts of dietary niacin. As of now, dietary niacin is not known to cause flushing or any other side effects that supplemental niacin can cause. Average intake of dietary niacin fluctuates from different populations depending on diet; a study on the population of Spain showed that their average daily intake was around 29.1 mg; where another study from China showed that a daily amount of niacin over 15.6 mg could cause hypertension [51,57]. These amounts reach nowhere near the amounts of supplemental niacin used in cardiovascular disease, which can be up into thousands of milligrams per day (Table 1).

## 4. Discussion

Niacin, commonly known as vitamin B3, has been an important part of cardiovascular disease management, particularly in the context of stroke prevention. As mentioned previously, niacin has a primary role in modulating lipid profiles by increasing high-density lipoprotein cholesterol, reducing low-density lipoprotein cholesterol, and lowering triglycerides. These changes were thought to contribute significantly to reducing atherosclerosis, which is a major risk factor for ischemic stroke. However, recent evidence from large-scale clinical trials have cast doubt on this long-term (>2 years) efficacy of niacin in reducing major cardiovascular events such as stroke [56]. This evidence suggests that while niacin favorably modifies lipid profiles, it does not consistently lead to reductions in stroke risk, questioning its role in contemporary stroke prevention strategies. There is a shift in understanding which represents the need for more detailed and targeted research to clarify niacin’s true impact on stroke prevention and its mechanisms.

There is an evolving landscape of cardiovascular research, highlighting areas where further studies are essential. Although prior research discussed niacin’s general mechanisms, the precise mechanisms by which niacin influences vascular health, particularly in its anti-inflammatory properties and its role in endothelial function, remain inadequately understood. Although niacin has also been shown to enhance NO production and reduce oxidative stress, the extent to which these effects translate into tangible clinical benefits, such as stroke prevention, is also not well defined. Future research should aim to unravel these mechanisms in greater detail by potentially identifying new therapeutic targets or biomarkers that could predict patient responses to niacin therapy. Another essential area of research is the differential response to niacin among various patient populations. Current evidence suggests that while some individuals experience significant benefits from niacin therapy, others do not, and some may even have experiences with adverse effects. Understanding the factors that contribute to these varying responses such as genetic predisposition, lifestyle factors, or underlying health conditions can help tailor niacin use more effectively. Few genetic variations of niacin response are known, but one includes a defect in the DGAT2 gene, involved in triglyceride synthesis, which may lead to a decreased lipid response to niacin supplementation [59]. More genetic variants and ethnic predispositions could be identified that could affect whether or not niacin could be effective in supplementation. The identification of genetic markers and ethnic predispositions could provide a framework for personalized niacin therapy, minimizing unnecessary exposure for non-responders. The safety profile of niacin in high doses is another critical area for future research. Understanding the long-term effects of niacin especially in the context of stroke prevention is vital. Research should aim to establish the optimal dosing regiments that balance efficacy with safety, particularly in long-term use. High-dose niacin is associated with adverse effects such as flushing, gastrointestinal discomfort, and dizziness, which may severely impact patients’ adherence. These real-world challenges must be addressed if niacin is to be seriously considered for stroke prophylaxis. The importance of this research extends beyond the academic community because it directly impacts public health strategies for stroke prevention. This makes effective prevention strategies a top priority. Given its potential risk in modulating risk factors for stroke, niacin could still be a valuable component of a multifaceted approach to stroke prevention, particularly if its use can be optimized based on the latest scientific evidence.

It also must be considered that the side effects of niacin usage may cause real-world challenges involving patient adherence and their overall quality of life. The side effects of niacin that could cause a patient to discontinue their supplementation include flushing, GI distress, and dizziness that could certainly impair their everyday lives. Given the high prevalence of these side effects, especially at therapeutic doses, clinicians must carefully weigh the benefits against the potential harm and patient nonadherence when considering niacin for stroke prevention. The prevalence of these side effects at high-dosage supplementation further warrants the justification of frequent niacin supplementation for stroke prevention.

In summary, the traditional role of niacin in stroke prevention has been challenged with recent evidence critical of benefits and important research gaps that need to be addressed. Historically, niacin was valued for lipid-modulating properties, which were believed to reduce the risk of atherosclerosis and stroke. However, the lack of consistent evidence supporting effectiveness in lowering stroke risk calls for a re-evaluation of its clinical role in the future. Current large-scale trials such as HPS2-THRIVE have demonstrated that despite improvements in lipid levels, niacin does not significantly reduce stroke incidence when added to statin therapy. Therefore, niacin should not be routinely recommended for stroke prophylaxis. Its use should be reserved for select patient populations with specific lipid abnormalities unresponsive to other therapies, and only after a thorough assessment of individual benefit–risk profiles.

The findings from recent trials underscore the complexity of niacin’s actions on cardiovascular health. The results suggest that while niacin can significantly alter lipid profiles, the expected reduction in stroke does not consistently follow, which illustrates that other factors play important roles. Addressing the inconsistencies through targeted research could lead to a more nuanced understanding of niacin’s role, which would enable healthcare providers to make more informed decisions about its use.

The significance of this research lies in its potential to refine clinical guidelines and improve patient outcomes. By filling the identified gaps, future studies could enhance the understanding of niacin benefits and limitations, which would lead to more effective and safer stroke prevention strategies. This could also lead to a broader understanding of how lipid management intersects with other aspects of cardiovascular health, providing insight that extends beyond niacin itself.

## 5. Conclusions

In conclusion, while niacin has played a historical role in stroke prevention, the emerging evidence necessitates a thorough re-evaluation of its use. Future research should focus on the underlying mechanisms, population-specific effects, and safety profiles. This approach would not only enhance the understanding of niacin’s role in vascular health but also improve stroke prevention strategies. This would ultimately benefit a wide range of patients.

## Figures and Tables

**Table 1:** Adverse effects of niacin: possible increase to stroke risk.

Adverse Effects of Niacin: Possible Increase to Stroke Risk			
Author	Groups Studied and Intervention	Results and Findings	Conclusions
Zhang Z et al. 2021. [51]	Dietary intake of niacin in Chinese adults.	J-shaped association with dietary niacin and new-onset hypertension, with the inflection point at 15.6 mg/d.	While niacin has beneficial vasodilatory properties, it is possible too much daily dietary niacin can cause new-onset hypertension not seen in supplemental niacin.
HPS2-THRIVE Collaborative. 2014. [56]	Different types of niacin therapy in adults with pre-existing cardiovascular disease.	While niacin therapy increased HDL and decreased LDL, niacin-laropiprant was associated with increase incidences of decreased diabetes control, increased diabetes diagnoses, increased serious adverse events in the gastrointestinal system and musculoskeletal system, increased infection, increased bleeding risk.	Adding niacin plus laropiprant to a statin-based therapy did not decrease the risk of major vascular events in patients with pre-existing cardiovascular disease but increased the risk of serious adverse events.
AIM-HIGH Investigators. 2011. [39]	Patients over 45 years old with pre-existing cardiovascular disease and low-baseline HDL and LDL levels. These patients had Niacin added to their Simvastatin regimen to determine if niacin had any cardiovascular significance.	Among these patients there was no significant benefit to adding niacin to the statin therapy, even though there were improvements to HDL cholesterol and triglycerides. There was also an unexpected rise in the number of ischemic strokes in the niacin-added group.	The overall rate of strokes was low in the niacin group but the number was higher than that of the placebo group. While no evidence can be found from this study about the correlation between niacin and ischemic stroke, there is now reason for this finding to be examined further in the future.
Koh Y et al. 2014. [58]	The effect of niacin therapy in sedentary nondiabetic postmenopausal women.	Niacin significantly increased glucose, insulin, and C-peptide levels.	While it has been known that niacin has tolerable effects in certain populations, this study shows that niacin use in certain populations can have more serious effects. When prescribing niacin to different populations, caution should be taken.

## Data Availability

No new data was created.
